# Ultraviolet-B Radiation (UV-B) Relieves Chilling-Light-Induced PSI Photoinhibition And Accelerates The Recovery Of CO_2_ Assimilation In Cucumber (*Cucumis sativus* L.) Leaves

**DOI:** 10.1038/srep34455

**Published:** 2016-09-30

**Authors:** Zi-Shan Zhang, Li-Qiao Jin, Yu-Ting Li, Mikko Tikkanen, Qing-Ming Li, Xi-Zhen Ai, Hui-Yuan Gao

**Affiliations:** 1 State Key Lab of Crop Biology, Tai’an, Shandong Province, China; 2 College of Horticulture Science and Engineering, Shandong Agricultural University, Tai’an, 271018, China; 3 College of Life Sciences, Shandong Agricultural University, Tai’an, 271018, China; 4 Plant Physiology and Molecular Biology, Department of Biochemistry and Food Chemistry, University of Turku, FIN–20014 Turku, Finland

## Abstract

Ultraviolet-B radiation (UV-B) is generally considered to negatively impact the photosynthetic apparatus and plant growth. UV-B damages PSII but does not directly influence PSI. However, PSI and PSII successively drive photosynthetic electron transfer, therefore, the interaction between these systems is unavoidable. So we speculated that UV-B could indirectly affect PSI under chilling-light conditions. To test this hypothesis, the cucumber leaves were illuminated by UV-B prior or during the chilling-light treatment, and the leaves were then transferred to 25 °C and low-light conditions for recovery. The results showed that UV-B decreased the electron transfer to PSI by inactivating the oxygen-evolving complex (OEC), thereby protecting PSI from chilling-light-induced photoinhibition. This effect advantages the recoveries of PSI and CO_2_ assimilation after chilling-light stress, therefore should minimize the yield loss caused by chilling-light stress. Because sunlight consists of both UV-B and visible light, we suggest that UV-B-induced OEC inactivation is critical for chilling-light-induced PSI photoinhibition in field. Moreover, additional UV-B irradiation is an effective strategy to relieve PSI photoinhibition and yield loss in protected cultivation during winter. This study also demonstrates that minimizing the photoinhibition of PSI rather than that of PSII is essential for the chilling-light tolerance of the plant photosynthetic apparatus.

Ultraviolet-B radiation (UV-B) is a common abiotic stress that restrains leaf expansion, Rubisco activity and the stomatal opening to reduce carbon assimilation and vegetative growth^1^,^2^. UV-B also damages DNA and proteins, particularly the photosynthetic pigment-protein complex in the thylakoid membrane^3^. The filtration of UV-B markedly increases the biomass of plants, but additional UV-B impairs the growth of plants^2^,^4^. Consequently, UV-B is generally considered to negatively impact plants^2^,^4^, although some research has shown that UV-B can suppress insect predation and pathogen infection in plants^5^,^6^,^7^.

However, this opinion considered only the Calvin cycle and photosystem II (PSII), while neglecting photosystem I (PSI) and the interaction between PSII and PSI. Although UV-B does not directly influence PSI^8^,^9^, PSI and PSII successively drive electron transfer in C3 plants; therefore, the interaction between them is unavoidable^10^. PSI is more stable than PSII under most abiotic stresses, such as high-light and high-temperature conditions^9^,^11^. However, when chilling-sensitive plants experience chilling-light stress^12^,^13^,^14^,^15^, or under the sequence of saturation pulses^16^,^17^,^18^, PSI becomes the primary photoinhibition site. Unlike PSII, which can quickly recoversfrom damage^18^,^19^,^20^, the recovery of the damaged PSI is quite slow and may be irreversible, even under optimum conditions^13^,^20^.

The electron transfer from PSII has been shown to be necessary for PSI photoinhibition^13^,^14^,^15^,^21^. Specifically, blocking the electron transfer from PSII to PSI with DCMU prevents the PSI photoinhibition induced by chilling-light conditions or fluctuating light^13^,^22^. During the recovery from chilling-light stress, the limited electron transport from PSII to PSI protects PSI from further photoinhibition and accelerates the recovery of PSI^13^.

Recent studies have identified several effective mechanisms that control the electron transport from PSII to PSI in C3 leaves^23^,^24^. The phosphorylation of PSII light-harvesting antenna (LHCII) balances the excitation of PSI and PSII and maintains the low reducing state of intersystem electron transfer chain under low-light conditions, which avoids excess electron transfer to the PSI acceptor side when the light intensity suddenly increases^25^. In addition, the protection of PSI from photodamage upon a shift from low light to high light requires a PGR5-dependent cyclic electron flow around PSI (CEF) that controls the speed of intersystem electron transfer via the Cyt b6f complex in the high light phase^22^,^26^,^27^,^28^. The ΔpH-dependent regulation of the Cyt b6f complex can slow the electron transfer from the plastoquinone (PQ) pool to plastocyanin (PC), avoiding excess electron transfer to PSI under high-light conditions^29^. Tikkanen *et al*.^30^ reported that the inhibition of de novo D1 synthesis protects PSI from photodamage in a *pgr5* mutant, which indicates that the PSII photoinhibition-repair cycle is an active regulatory component of the photosynthetic electron transfer to PSI. Although the sustained non-photochemical quenching (NPQ) that limits the light energy capture of PSII helps to maintain PSI activity in overwinter plants during harsh winters^31^,^32^,^33^,^34^, the NPQ adversely affects PSI in other species. Recent research showed that the deletion of PsbS protein exacerbates PSII photoinhibition but alleviates PSI photoinhibition under high-light conditions in *Arabidopsis thaliana*^35^ and *Oryza sativa* L.^36^.

UV-B does not directly influence PSI but significantly inhibits PSII^37^. PSI and PSII successively drive photosynthetic electron transfer, therefore, the interaction between these systems is unavoidable. So we speculated that UV-B could indirectly affect PSI under chilling-light conditions. Here, we provide evidence to show that UV-B controls the electron transfer to PSI by restraining the activity of the oxygen-evolving complex (OEC), which ultimately protects PSI from photoinhibition under chilling-light stress. After chilling-light stress, the recovery of PSI activity and carbon assimilation capacity were accelerated in UV-B exposed leaves, which should attenuate the loss of yield and growth of cucumbers caused by chilling-light stress.

## Results

### The influence of UV-B on PSI and PSII activities

**Figure 1:**
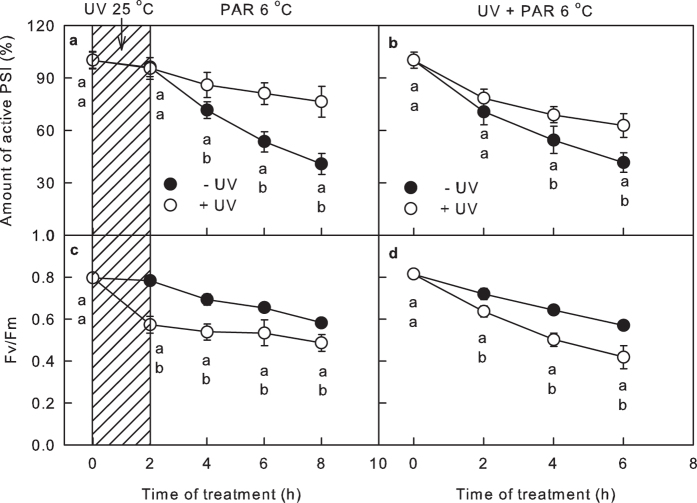
The influence of UV-B to PSI and PSII photoinhibition under chilling-light condition. The activity of the PSI complex and the maximum quantum yield of PSII (Fv/Fm) in leaves exposed to chilling-light and UV-B conditions for the indicated times. In plots (**a**,**c**), the leaves were exposed to approximately 6 μmol m^−2^ s^−1^ UV-B (+UV) or darkness (−UV) at 25 °C for 2 h (shaded part), and the leaves were then transferred to chilling-light conditions (6 °C, 200 μmol m^−2^ s^−1^) for 6 h. In plots (**b**,**d**), the leaves were exposed to chilling-light conditions (6 °C, 200 μmol m^−2^ s^−1^) in the presence (+UV) and absence (−UV) of approximately 2 μmol m^−2^ s^−1^ UV-B for 6 h. The means ± SE, n = 12. Different letters indicate significant differences between +UV and −UV treated leaves at P < 0.05.

Exposure to UV-B at normal temperatures did not markedly affect the activity of the PSI complex but steeply decreased the maximum quantum yield of PSII (Fv/Fm) (Fig. 1a,c). During the subsequent chilling-light treatments, the Fv/Fm was consistently lower in leaves pre-exposed to UV-B than in leaves that had not been pre-exposed to UV-B. Interestingly, UV-B pretreatment markedly alleviated the decrease in the activity of the PSI complex during the chilling-light treatment (Fig. 1a,c). In another experiment, the leaves were irradiated with weaker UV-B during chilling-light treatment; PSI photoinhibition was also alleviated in this experiment, and the PSII photoinhibition was more severe (Fig. 1b,d).

**Figure 2:**
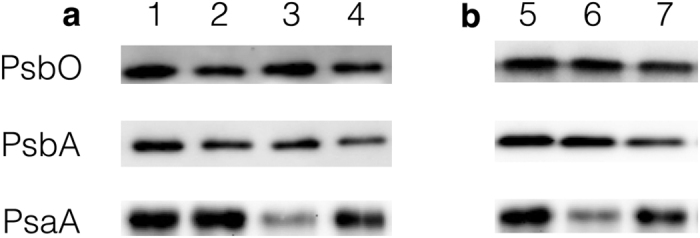
Immunoblot analysis of thylakoid membranes proteins extracted from leaves. Line 1, the leaves were exposed to darkness at 25 °C for 2 h; line 2, the leaves were exposed to approximately 6 μmol m^−2^ s^−1^ UV-B at 25 °C for 2 h; line 3, the leaves were exposed to darkness at 25 °C for 2 h and the leaves were then transferred to chilling-light conditions (6 °C, 200 μmol m^−2^ s^−1^) for 6 h; line 4, the leaves were exposed to approximately 6 μmol m^−2^ s^−1^ UV-B at 25 °C for 2 h and the leaves were then transferred to chilling-light conditions (6 °C, 200 μmol m^−2^ s^−1^) for 6 h; line 5, before treatment; line 6, the leaves were exposed to chilling-light conditions for 6 h; line 7, the leaves were exposed to chilling-light conditions in the presence of approximately 2 μmol m^−2^ s^−1^ UV-B for 6 h; Thylakoid membranes (10 μg of chlorophyll) were separated by SDS-PAGE, electroblotted, and probed using specific antibodies against PsbO, PsbA, and PsaA.

The above results were verified by the following immunoblot analysis. Treatment with UV-B before or during the chilling-light treatment decreased the amount of PSII protein (PsbO and PsbA) but prevented the decrease in PSI protein (PsaA) during chilling-light treatment (Fig. 2).

**Figure 3:**
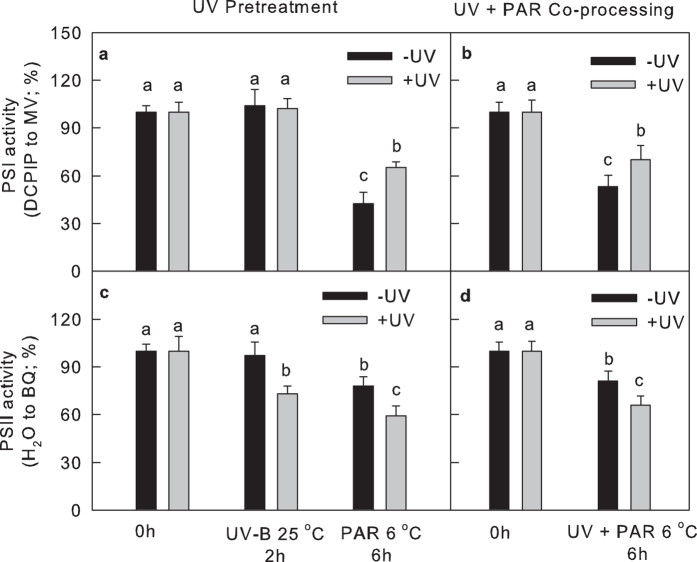
The PSI (DCPIP-MV) and PSII (H_2_O-BQ) electron transport activities of isolated thylakoid membranes in leaves exposed to chilling-light treatment and UV-B. In plots (**a**,**c**), the leaves were exposed to approximately 6 μmol m^−2^ s^−1^ UV-B (+UV) or darkness (−UV) at 25 °C for 2 h, and the leaves were then transferred to chilling-light conditions (6 °C, 200 μmol m^−2^ s^−1^) for 6 h. In plots (**b**,**d**), the leaves were exposed to chilling-light (6 °C, 200 μmol m^−2^ s^−1^) in the presence (+UV) or absence (−UV) of approximately 2 μmol m^−2^ s^−1^ UV-B for 6 h. The means ± SE, n = 12. Different letters indicate significant differences between +UV and −UV treated leaves at P < 0.05.

To study the effect of UV-B to the capacity of electron transfer from PSII to PSI, we next analysed the electron transport rate in the thylakoid membrane. UV-B markedly decreased the electron transfer capacity of PSII to the acceptor side (H_2_O to BQ). During chilling-light treatment, the capacity of electron transfer from donor side to acceptor side of PSI (DCPIP to MV) was protected from decreases by UV-B irradiation or UV-B pre-irradiation (Fig. 3).

The above results clearly demonstrated that UV-B protected PSI from chilling-light-induced photoinhibition by inactivating PSII and decreasing the electron transfer from PSII to PSI.

### The influence of UV-B on the donor and acceptor sides of PSII

**Figure 4:**
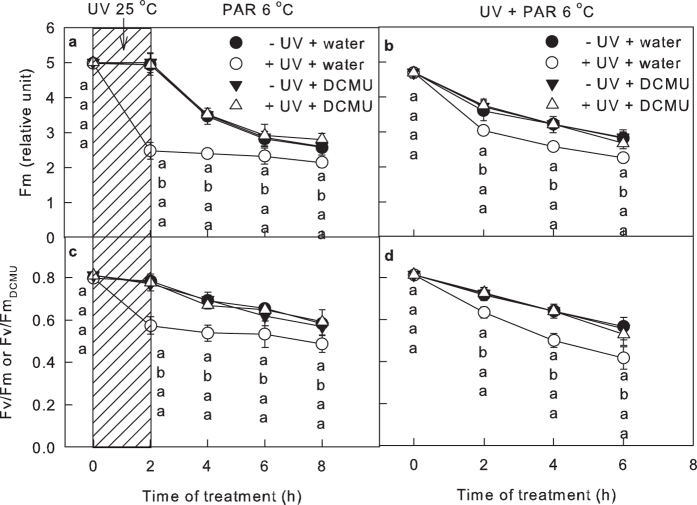
The maximum fluorescence (Fm or Fm_DCMU_; (**a**,**b**)) and maximum quantum yield of PSII (Fv/Fm or Fv/Fm_DCMU_; (**c**,**d**)) in leaves exposed to chilling-light and UV-B conditions for the indicated times. In plots (**a**,**c**), the leaves were exposed to approximately 6 μmol m^−2^ s^−1^ UV-B (+UV) or darkness (−UV) at 25 °C for 2 h (shaded part), and the leaves were then transferred to chilling-light conditions (6 °C, 200 μmol m^−2^ s^−1^) for 6 h. In plots (**b**,**d**), the leaves were exposed to chilling-light conditions (6 °C, 200 μmol m^−2^ s^−1^) in the presence (+UV) or absence (−UV) of approximately 2 μmol m^−2^ s^−1^ UV-B for 6 h. After chilling-light and UV-B treatment, the leaves were infiltrated with DCMU or water in the dark for 1 h. The original Fm or the Fm measured in the presence of DCMU (Fm_DCMU_) was used to calculate Fv/Fm (or Fv/Fm_DCMU_). The means ± SE, n = 12. Different letters indicate significant differences between +UV and −UV treated leaves at P < 0.05.

The immunoblot analysis (Fig. 2) showed that UV-B damaged both the core protein of oxygen-evolving complex (OEC; PsbO) and Q_B_-banding protein (PsbA), which may suppress the electron support at the PSII donor side and electron transfer from Q_A_ to Q_B_ at PSII acceptor side, respectively. Although the decrease in Fv/Fm has been used to reflect PSII photoinhibiton, both the donor side inactivation and acceptor side inactivation have been proven to decrease the Fv/Fm^13^,^38^,^39^,^40^. Thus, to compare the inactivation of the donor and acceptor sides of PSII after UV-B exposure, leaves exposed to chilling-light (−UV leaves) or chilling-light-UV-B (+UV leaves) conditions for 2 h were infiltrated with DCMU or water in the dark, and the maximum fluorescence (Fm) was then measured with a saturated flash (Fig. 4).

DCMU did not influence the Fm in −UV leaves, which indicates that a flash of light can completely reduce Q_A_^−^, irrespective of the presence of DCMU (Fig. 4). However, DCMU markedly enhanced the Fm in +UV leaves (Fig. 4), which indicates that the Q_A_ cannot be completely reduced by a flash in +UV leaves without the presence of DCMU. This effect is attributed to that the electron transfer from OEC to Q_A_ was too weak to overcome the electron transfer beyond Q_A_. To avoid the underestimation of Fm and Fv/Fm due to the inactivation of OEC, the Fm measured in the presence of DCMU (Fm_DCMU_) was used instead of the original Fm to calculate Fv/Fm (Fv/Fm_DCMU_). The Fv/Fm_DCMU_ was higher than original Fv/Fm in +UV leaves, but the Fv/Fm_DCMU_ was similar in +UV and −UV leaves (Fig. 4c,d). These results proved that in UV-exposed leaves, the inactivation of OEC, rather than the inhibition of electron transfer from Q_A_ to Q_B_, dominated the electron transfer from PSII to PSI and PSI photoprotection.

### The influence of UV-B on the recovery of the photosynthetic apparatus

**Figure 5:**
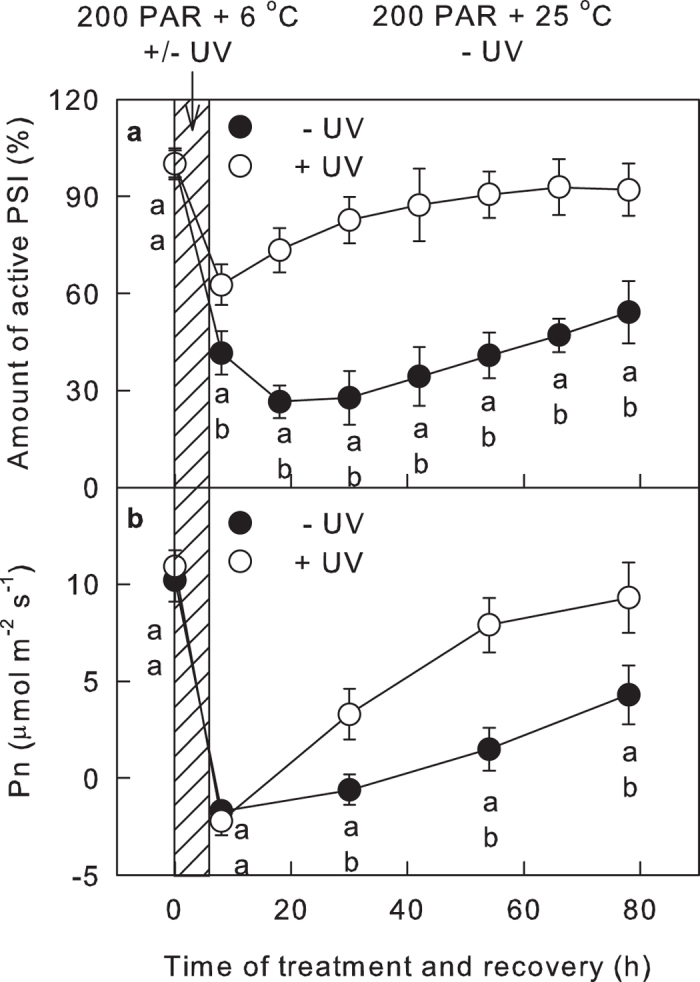
UV-B during chilling-light treatment accelerated the following recovery of the activity of the PSI complex and photosynthetic rate. The activity of the PSI complex (**a**) and photosynthetic rate (Pn; (**b**)) in leaves exposed to chilling-light conditions in the presence (+UV) or absence (−UV) of approximately 2 μmol m^−2^ s^−1^ UV-B for 6 h (shaded part) and subsequent repair process at 25 °C under low-light conditions (15 μmol m^−2^ s^−1^) for 72 h. The means ± SE, n = 12 (**a**) or 6 (**b**). Different letters indicate significant differences between +UV and −UV treated leaves at P < 0.05.

The eventual effect of abiotic stress on the photosynthesis and growth of plants is not only a result of the extent of the damage to the photosynthetic apparatus but also depends on the capacity for recovery after the damage has taken place^13^,^41^. Thus, leaves exposed to chilling-light (−UV leaves) or chilling-light-UV-B (+UV leaves) conditions for 6 h were transferred to normal temperature and low-light conditions (25 °C and 15 μmol m^−2^ s^−1^ PAR) for recovery. The activity of the PSI complex in +UV leaves recovered to approximately 87% of the initial activity within 36 h (Fig. 5a). In sharp contrast to +UV leaves, the activity of PSI complex in −UV leaves did not recover in the first 36 h of the recovery process; it recovered to less 50% of the initial activity after 72 h (Fig. 5a). Next, the photosynthetic rate (Pn) during the stress and recovery process was also investigated. Six hours of chilling-light stress decreased the Pn to negative values, irrespective of the presence of UV-B (Fig. 5b). However, during the subsequent recovery process, the recovery of Pn was much faster in +UV leaves than in −UV leaves (Fig. 5b).

## Discussion

The PSI photoinhibition depends on the balance between the electron transfer to the acceptor side of PSI and the consumption of electrons by the Calvin cycle and other metabolic processes. Chilling stress causes massive electron transfer to O_2_ due to the severe inhibition of Calvin cycle^21^,^42^. Chilling inactivated the thylakoid-bound ascorbate peroxidases and superoxide dismutase, which caused the accumulation of ROS^42^,^43^. ROS invariably damage the neighbouring iron-sulphur cluster and PsbA/B protein. In chilling-sensitive species, the Calvin cycle and ROS-scavenging enzymes are more sensitive to cold than in other species^10^,^21^. Thus, limiting electron transfer to PSI is critical to avoiding PSI photoinhibition caused by chilling-light stress in chilling-sensitive species.

**Figure 6:**
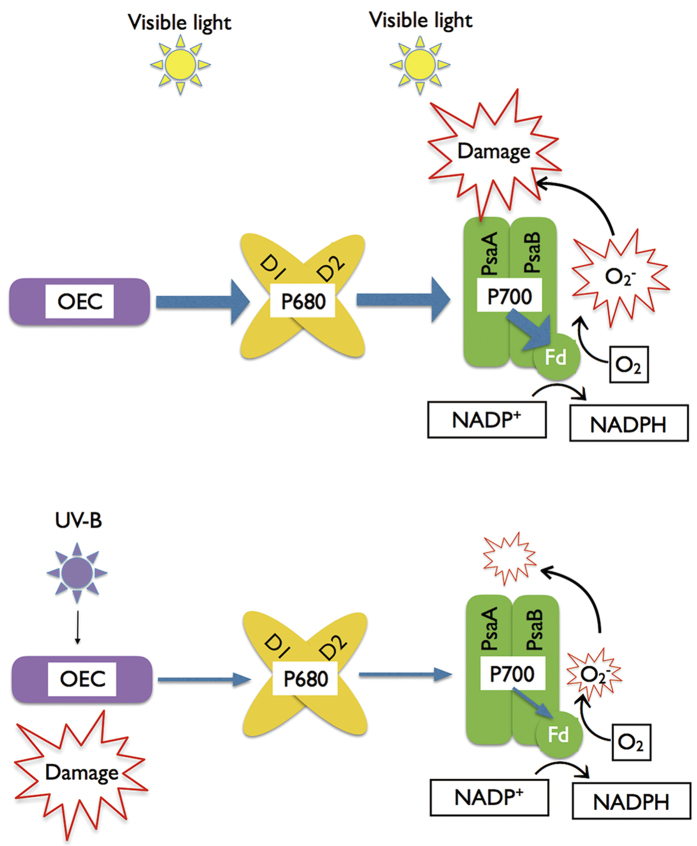
Scheme of UV-B protects PSI from photoinhibition. OEC, oxygen-evolving complex; Fd, ferredoxins.

Previous studies reported that the mechanisms contributing to the control of electron transport to PSI involve LHCII phosphorylation^25^, the ΔpH-29, CEF-dependent control of Cyt b6f^22^,^26^, and the dynamic regulation of D1 protein turnover^30^. The LHCII phosphorylation is only operational under fluctuating light, but this study examined only constant light conditions. Although D1 protein degradation (Figs 1,2 and 3), PGR5-dependent CEF^13^, and ΔpH dependent NPQ^44^,^45^,^46^ have been shown to be enhanced in cucumber leaves under chilling-light stress, however, PSI photoinhibition was not prevented (Figs 1,2 and 3). These factors indicate that the above mechanisms were not sufficiently strong to limit electron flow to PSI and thereby protect PSI in cucumber leaves under chilling-light stress. Here, we proved that UV-B inactivate OEC and thereby limited the photosynthetic electron flow to PSI (Fig. 6). Due to sunlight contains both UV-B and PAR, we suggest that the UV-B-induced PSII inactivation is critical for PSI photoprotection, irrespective of dynamic or constant light conditions. Because the OEC activity cannot immediately recover, the UV-B-induced OEC inactivation could protect PSI in the long term.

Tikkanen *et al*.^30^ reported that pre-illumination in the presence of lincomycin protected PSI from photoinhibition during subsequent high-light treatment. The presence of Lin and pre-illumination resulted in the degradation of D1 protein, which inhibited the electron transfer at the acceptor side of PSII. In contrast, this study proved that in UV-exposed leaves, the inactivation of OEC, rather than the inhibition of electron transfer from Q_A_ to Q_B_, caused the increased electron transfer from PSII to PSI (Figs 2 and 4). Consequently, the dynamic regulation of D1 protein turnover and the sensitivity of OEC to UV-B are distinct mechanisms for the control of electron transfer to PSI. It has been reported that after the cucumber leaves were pretreated by dark-chilling, which inactivating the OEC, the PSI was protected from photoinhibition under following chilling-light condition^41^.

The entire photosynthetic apparatus, including the dark and light reactions, was inhibited by chilling-light stress, but the activities of ATPase, Calvin-cycle enzymes and PSII were restored during the recovery process^47^,^48^,^49^. PSI is the limiting factor of the recovery of Pn after chilling-light stress^13^,^20^. Consequently, the prevention of PSI photoinhibition and the acceleration of recovery in PSI activity are essential for the recovery of Pn after chilling-light stress.

Although UV-B damaged PSII (Figs 1,2 and 3), suppressed the opening of stoma and inhibited the Calvin-cycle^1^,^2^, this study showed that these negative effects minimally influenced the photosynthesis rate (Fig. 5). However, the photoprotective effect of UV-B on PSI improved the recovery of Pn. Irreversible decreases in Pn result in a loss of yield during and after chilling-light stress, and additional UV-B irradiation can effectively prevent this loss to protect cultivation during winter. In contrast, the other mechanisms that control electron transport to PSI, such as LHCII phosphorylation and the ΔpH- and PGR5-dependent control of Cyt b6f, are complex, and their regulatory pathways are poorly understood. In addition, these mechanisms are multifunctional, and artificially modifying them may result in metabolic disturbances in cells. Consequently, these mechanisms cannot currently be used to improve the cold tolerance of PSI and Pn, whereas UV-B can be used to this end, as demonstrated in this study.

## Conclusion

Previously, UV-B was considered to negatively impact the photosynthesis and growth of plants. However, this study demonstrated that UV-B decreased the electron transfer to PSI by inactivating the OEC, thereby protecting PSI from chilling-light-induced photoinhibition. This effect advantages the recovery of Pn after chilling-light stress and minimized yield losses caused by chilling-light stress. This study also demonstrated that minimizing the photoinhibition of PSI rather than that of PSII is essential for the cold-tolerance of the photosynthetic apparatus. In addition, in contrast to sunlight, the artificial light source used in photosynthetic research does not contain UV-B, which may increase PSI photoinhibition. Thus, a light spectrum close to that of solar radiation spectrum should be considered in future studies.

## Methods

### Plant materials and growth conditions

Cucumber (*Cucumis sativus* L. cv. Jinyou 3) plants were grown in pots (7 cm in diameter, 10 cm in height) filled with rich soil. This rich soil supplied sufficient nutrients to plants, and the plants were supplied with sufficient water to grow. The plants were placed in a growth chamber at 25/22 °C and 150 μmol m^−2^ s^−1^ with a 14 h/10 h photoperiod. The youngest fully developed leaves of approximately 4-week-old plants were used in the experiments.

### Treatments

**Figure 7:**
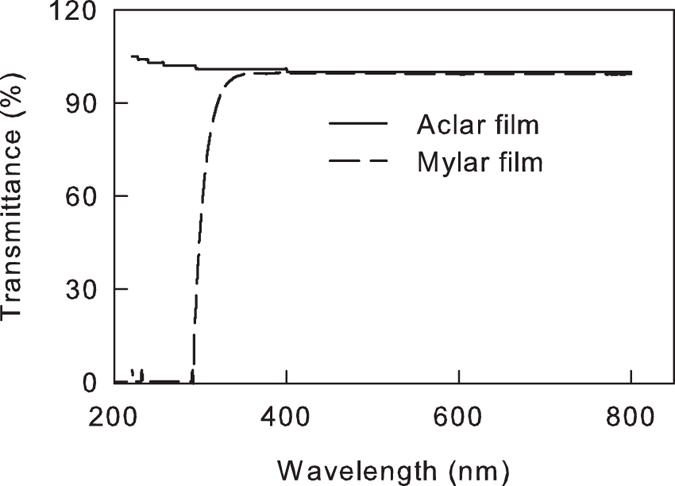
Spectral transmissions of the two spectral filters used in this study. Mylar film excluding most UV-B or Aclar film characterized by high UV-B transmittance. The variation between multiple samples of identical films was negligible, and the SEs are too small to present.

The abaxial sides of attached leaves of the cucumber plants were placed on the surface of water at 6 °C. The temperature of the water was controlled by an RTE-211 water circulator (Thermo, USA). The leaves were illuminated by photosynthetically active radiation (PAR) and UV-B, which were obtained from a red and blue (8:1) light-emitting diode (Senpro, China) and a PL-S 9W/01/2P UV-B light source (Philips, Netherlands), respectively. The leaves were separated with PAR and UV-B light source by a 0.13 mm polyester plastic film (Mylar D; DuPont, USA) that excluded most UV-B or a 0.08-mm Aclar film (22A; Allied Signal, USA), which is characterized by a high transmittance of UV-B. The films were placed on 5 cm above the leaves. The spectral transmittance of the filters was measured using a spectrophotometer UV-1601 (Shimadzu, Japan; as shown in Fig. 7). The transmittance of PAR (400–700 nm) is high in both films (Fig. 7). The intensities of PAR and UV-B on the surface of leaves were adjusted by altering the distance between the surface of the leaf and light source. A 3414F LightScout UV meter (Spectrum, USA) or a 3415F LightScout quantum meter (Spectrum, USA) were used to measure the intensity of UV-B or PAR radiation.

Two series UV-B treatments were performed in this study. Series 1 is the UV-B pre-treatment experiment, in which the leaves were exposed to approximately 6 μmol m^−2^ s^−1^ UV-B or darkness at a normal temperature (25 °C) for 2 h. The leaves were then transferred to chilling-light conditions (200 μmol m^−2^ s^−1^ PAR and 6 °C) for 6 h. Series 2 refers to the UV-B and PAR co-processing experiment, in which the leaves were exposed to chilling-light conditions (200 μmol m^−2^ s^−1^ PAR and 6 °C) in the presence or absence of approximately 2 μmol m^−2^ s^−1^ UV-B for 6 h. The intensity of UV-B used in series 2 was lower than it in series 1 due to the treatment period in series 2 was much longer than it in series 1.

For inhibitor treatment, the leaves were submerged in 70 μM 3-(3,4-Dichlorophenyl)-1,1-dimethylurea (DCMU) or water for 1 h in the dark and then placed in air for 20 min before further experiments.

### Measurements of the chlorophyll a fluorescence transient (OJIP) and 820-nm transmission

The Chl a fluorescence transient, and the 820-nm transmission were synchronously measured using an integral Multifunctional Plant Efficiency Analyser (M-PEA; Hansatech, UK) with dark-adapted leaves at 25 °C. A 2 s saturating red light pulse of 5000 μmol m^−2^ s^−1^ produced by an array of four light-emitting diodes (peak 650 nm) was used in the measurements. The maximum quantum yield of PSII (Fv/Fm) was calculated with the following equation: Fv/Fm = 1−(Fo/Fm). The initial slope of the oxidation phase indicates the capability of P700 to be oxidized, which was used to reflect the activity of the PSI complex^50^,^51^,^52^,^53^,^54^.

### Isolation of Thylakoid Membranes

Five grams of leaf discs were homogenized on ice in 400 mM sucrose, 50 mM HEPES-KOH (pH 7.8), 10 mM NaCl, and 2 mM MgCl_2_ and filtered through two layers of cheesecloth. The resulting filtrates were centrifuged at 5000 g for 10 min at 4 °C, and the thylakoid pellets were resuspended in the homogenization buffer. Chlorophyll content was determined according to the method of^55^.

### SDS-PAGE and Immunoblot analysis

Thylakoid membranes (10 μg chlorophyll) were loaded on and separated by a 12% (w/w) SDS-PAGE gel. Proteins from the gel were subsequently blotted onto nitrocellulose by standard methods. The blot was blocked with 5% (w/w) skimmed milk and then incubated for 2 h with the primary antibody. Subsequently, the blot was incubated with horseradish peroxidase-conjugated anti-rabbit IgG antibody (Solarbio, Beijing, China) for 2 h. The Supersignal West Pico substrate (Thermo Fisher Scientific, Shanghai, China) was applied to detect the immunoreaction. The chemiluminescence was recorded on blots using a Tanon-5500 cooled CCD camera (Tanon, Shanghai, China). The primary anti-PsbO, anti-PsbA, anti-PsaA antibodies against synthetic polypeptides containing amino acid residues CQPSDTDLGAKVPKD, APPVDIDGIREPVSC, CRDYDPTTRYNDLLD, respectively, were purchased from Genscript company (Nanjing, China).

### Photosynthetic electron transport measurements

Electron transport was measured using a Clark-type oxygen electrode under 1000 μmol m^−2^ s^−1^ white light^56^. PSI electron transport was assayed in a 3 mL reaction mixture composed of 40 mM Tricine, pH 7.5, 10 mM Suc, 167 μM MV, 0.1 mM DCPIP. 1 mM sodium ascorbate, 10 mM NH_4_Cl, 10 μM DCMU, 5 mM sodium azide, and thylakoids corresponding to 15 μg of chlorophyll. Similarly, PSII electron transport was assayed in a 3 mL reaction mixture composed of 50 mM Tricine, pH 7.5, 20 mM NaCl, 5 mM MgCl_2_, 100 mM Suc, 1 mM phenyl-p-benzoquinone, and thylakoids corresponding to 15 μg of chlorophyll.

### Photosynthetic gas exchange measurements

The net photosynthetic rate (Pn) was measured using a CIRAS-3 portable photosynthesis system (PP Systems, USA) at 25 °C, 800 μmol m^−2^ s^−1^ light (90% red light and 10% blue light), 400 μmol mol^−1^ CO_2_ and 60–65% relative humidity. Leaf temperature, light intensity, CO_2_ concentration and relative humidity were controlled using the automatic control device of the CIRAS-3 portable photosynthetic system. After chilling-light treatment, the Pn were measured 2 h after the leaves were transferred from cool water to normal temperature.

## Additional Information

**How to cite this article**: Zhang, Z.-S. *et al*. Ultraviolet-B Radiation (UV-B) Relieves Chilling-Light-Induced PSI Photoinhibition And Accelerates The Recovery Of CO_2_ Assimilation In Cucumber (*Cucumis sativus* L.) Leaves. *Sci. Rep*. **6**, 34455; doi: 10.1038/srep34455 (2016).
